# Role of Cannabis in the Management of Chronic Non-Cancer Pain: A Narrative Review

**DOI:** 10.3390/clinpract15010016

**Published:** 2025-01-13

**Authors:** Lou’i Al-Husinat, Shrouq Obeidat, Saif Azzam, Yara Al-Gwairy, Fatima Obeidat, Sarah Al Sharie, Deema Haddad, Fadi Haddad, Martina Rekatsina, Matteo Luigi Giuseppe Leoni, Giustino Varrassi

**Affiliations:** 1 Department of Clinical Sciences, Faculty of Medicine, Yarmouk University, Irbid 21163, Jordan; loui.husinat@yu.edu.jo; 2 Faculty of Medicine, Yarmouk University, Irbid 21163, Jordan; anaobeidat1@gmail.com (S.O.); saifazzam2000@gmail.com (S.A.); yaraalghwairy@gmail.com (Y.A.-G.); depixag745@segichen.com (F.O.); haddad@yu.edu.jo (F.H.); 3 Office of Scientific Affairs and Research, King Hussein Cancer Center, Amman 11941, Jordan; 4 Faculty of Medicine, Jordan University of Science and Technology, Irbid 22110, Jordan; dwhaddad@med.just.edu.jo; 5 Department of Anesthesia, University of Athens, 11528 Athens, Greece; mrekatsina@gmail.com; 6 Department of Medical and Surgical Sciences and Translational Medicine, “La Sapienza” University of Rome, 00100 Rome, Italy; matteolg.leoni@gmail.com; 7 Fondazione Paolo Procacci, 00193 Roma, Italy; giuvarr@gmail.com

**Keywords:** chronic non-cancer pain, quality of life, pain management, cannabis, antipsychotic properties

## Abstract

Chronic non-cancer pain, defined by the Center for Disease Control and Prevention (CDC) as lasting beyond three months, significantly affects individuals’ quality of life and is often linked to various medical conditions or injuries. Its management is complex. Cannabis, containing the key compounds Delta-9-tetrahydrocannabinol (THC) and cannabidiol (CBD), has garnered interest for its potential in pain management, though it remains controversial due to its psychoactive effects and illegal status in many countries. THC provides pain relief by blocking nociceptive stimuli but has psychoactive effects and may potentially induce dependency. CBD has calming and antipsychotic properties. The inhalation of cannabis offers quick relief but poses respiratory risks, while its oral administrations are safer but act more slowly. Short-term cannabis use can impair cognition and motor skills, while long-term use may lead to dependency and cognitive decline, especially if used from an early age. Adverse effects vary by gender and prior use, with addiction mainly linked to THC and influenced by genetics. Despite these risks, patients often report more benefits, such as improved quality of life and reduced opioid use, although the evidence remains inconclusive. The legal landscape for medical cannabis varies globally, with some positive public health outcomes like reduced opioid-related issues in areas where it is legalized. Cannabis shows promise in managing chronic pain, but its psychoactive effects and dependency risks necessitate cautious use. Future research should prioritize long-term clinical trials to establish optimal dosing, efficacy, and safety, aiding in the development of informed guidelines for safe cannabis use in chronic pain management. This review examines the use of cannabis in managing chronic non-cancer pain, focusing on its benefits, drawbacks, mechanisms, delivery methods, and impact on quality of life.

## 1. Introduction

Chronic non-cancer pain has been defined by the Centers for Disease Control and Prevention (CDC) in 2016 as pain lasting longer than 3 months, or pain lasting beyond the scope of the healing of tissue [1]. It can be the result of a variety of medical conditions or injuries and might even be idiopathic [1]. The University of Düsseldorf analyzed a survey of 10,291 respondents in 2008. The results showed that the prevalence of chronic pain was 10.1% for pain originating from the back, 7.1% for lower limb pain, 4.1% for upper limb pain, and 3.5% for headache [2]. The 2012 National Health Interview Study contained data showing that 11.2% of adults have pain every day [3]. Chronic pain can severely impact an individual’s quality of life and, due to its multifaceted nature, can be a challenging condition to manage and treat [4]. A patient’s physical and mental health can be severely affected, as chronic pain is highly comorbid with depression and anxiety spectrum disorders, with the relationship between depression and pain often described as a two-way complex relationship [5]. This dysfunctional relationship can lead to a decline in psychological functioning as well as ineffective coping mechanisms, including catastrophizing, perceived helplessness, and low self-efficacy [6]. The CDC issued guidelines back in 2016 to try and minimize medical practitioners from overprescribing opioids to patients complaining of chronic pain in an attempt to mitigate the rising rates of opioid deaths in the US [7].

Cannabis is a cultivated plant. Its usage dates back to the 6th century B.C. [8], and it is currently considered the most commonly used illegal substance worldwide. In total, 80.5 million Europeans have used cannabis at least once in their lifetimes [9]. Cannabis, alongside Humulus, is a part of the Cannabaceae family [8]. Over 421 different substances have been discovered in cannabis, including flavonoids, nitrogenous compounds, amino acids, and sugar [10]. However, the two most important ingredients in cannabis which are responsible for most of its behavioral effects are Delta-9-tetrahydrocannabinol (THC) and Cannabidiol (CBD) [10]. THC is believed to be the primary psychoactive component, inducing psychosis, anxiety, and paranoia. It also has potent analgesic effects by blocking nociceptive stimuli [11,12]. THC is believed to be the cause of addiction and dependance on cannabis [11,12]. On the other hand, CBD has calming effects that combat anxiety, as well as antipsychotic effects, by enhancing glutamate activity in the prefrontal cortex [12]. The legalization of cannabis in the United States and several other countries is a controversial conflict that is yet to be resolved, leaving confusion in both physicians and patients alike. The first law allowing for the medical administration of cannabis was ruled in 1996. The administration of cannabis for medical purposes in a healthcare setting is currently considered legal in about 50% of the states in the USA [13], as well as in several other countries around the world [14,15,16,17,18,19,20].

The purpose of this review is to analyze the potential usage of cannabis in the management of chronic non-cancer pain, explaining its advantages and disadvantages, mechanisms of action, forms and routes of delivery, and its effect on a patient’s quality of life. We will provide a comprehensive resource to address future guidelines and studies for a safer, more effective approach to the management of chronic non-cancer pain.

## 2. Methodology

This narrative review employs a comprehensive descriptive thematic analysis to explore the role of cannabis in managing chronic non-cancer pain. This review adheres to the Scale for the Assessment of Narrative Review Articles (SANRA) standards to ensure methodological rigor and credibility in the scientific community [21].

### 2.1. Eligibility Criteria

Eligible studies were peer-reviewed empirical or prospective studies focused on cannabis and its role in chronic non-cancer pain management. The types of studies considered included original articles, reviews, case series, case reports, or letters to the editor, provided that the full text was accessible. Studies were excluded if they did not align with the designated topic, lacked an adequate reporting of their objectives and conclusions, or did not provide full-text access.

### 2.2. Information Sources and Search

A thorough literature search was conducted across multiple databases, including PubMed, Scopus, Embase, PsycINFO, Web of Science (WoS), and Google Scholar, to ensure comprehensive coverage of the existing literature. The search strategy utilized Medical Subject Heading (MeSH) terms tailored to capture the essence of the investigation, focusing on cannabis, chronic non-cancer pain, and related terms. Boolean operators “AND” and “OR” were used to refine the search. For instance, the primary search term was “cannabis AND chronic non-cancer pain”. Additionally, the search was expanded to include synonyms, variants, and alternative formulations like “pain management” AND “THC”.

### 2.3. Selection Process

Two independent authors screened titles and abstracts against the eligibility criteria, and this was followed by full-text assessments. Any discrepancies were resolved through discussion or consultation with a third reviewer. The initial pilot screenings achieved over 80% agreement, ensuring the reliability of the selection process.

## 3. Mechanism of Action

### The Endocannabinoid System

The mechanism of action for cannabis is dependent on the signaling pathways of THC and CBD. Murine neuroblastoma cells were found to increase the synthesis of adenylate cyclase when subjected to THC, which led to the isolation of a receptor called cannabinoid receptor type 1 (CB1) [22]. Another receptor, called cannabinoid receptor type 2 (CB2), was discovered later [22]. CB1 receptors are more commonly prevalent in the frontal cortex, hippocampus, basal ganglia, hypothalamus, cerebellum, and spinal cord [23]. Mapping studies on a rat brain showed that CB1 receptors are confined to the axons and nerve terminals away from the cell body [24]. CB2 receptors, on the other hand, are largely found on immune and hematopoietic cells [25]. CB1 receptors are suggested to have an effect on neurotransmitter release, inhibiting the release of L-glutamate, Gamma-aminobutyric acid (GABA), noradrenaline, dopamine, 5-hydroxytryptamine (5-HT), and acetylcholine. THC is theorized to activate CB1 receptors on GABA-containing neurons, decreasing the release of GABA and increasing the activity of the dopaminergic tract [26,27].

The discovery of cannabinoid receptors led to the discovery of several ligands and derivatives of arachidonic acid, which have significant effects on the receptors [28]. They were named Endogenous Cannabinoids, or endocannabinoids. The endocannabinoid system refers to the collective of endocannabinoids, cannabinoid receptors, and proteins recruited for the creation, destruction, and mobility of endocannabinoids [28]. 2-arachidonylglycerol (2-AG) and *N*-arachidonoylethanolamine (AEA) are the most notable endogenous cannabinoids [24]. Phospholipase C leads to the synthesis of diacylglycerol, which is then converted to 2-AG [29]. Different cannabinoid receptors show selectivity in their function and affinity to different endocannabinoids. This implies that multiple agonists different in structure and composition may elicit a plethora of various effects and physiological symptoms. CB1, for example, is highly activated by 2-AG compared to THC, as the effects of CB1 would be more prominently pronounced when bound to 2-AG [28,30].

**Figure 1:**
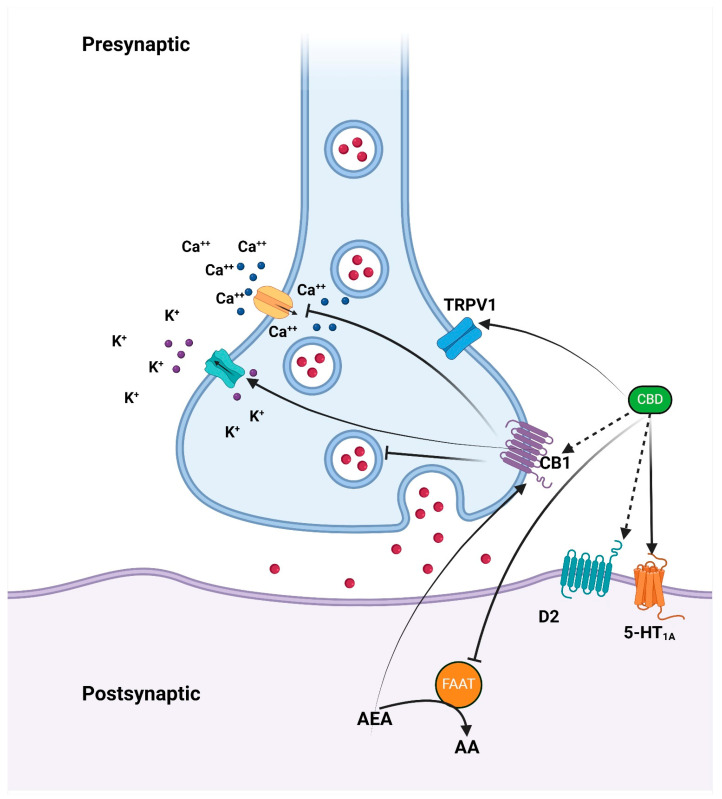
The mechanism by which CBD affects psychotic disorders involves the inhibition of FAAH, leading to elevated anandamide levels. Anandamide subsequently activates CB1, CB2, and TRPV1 receptors. Additionally, CBD can directly activate TRPV1 receptors. The partial agonism at the D2 dopamine receptors may explain CBD’s impact on emotional memory processing within the ventral hippocampus. Abbreviations: 5-HT1A (5-hydroxytryptamine 1A receptor), AEA (anandamide), CB1 (cannabinoid receptor 1), D2 (dopamine receptor 2), FAAH (fatty acid amide hydrolase), and TRPV1 (transient receptor potential vanilloid 1).

Endocannabinoids function as retrograde messengers, traveling to the pre-synapse to activate cannabinoid receptors [31]. They cause plasticity in the synapses through post-synaptic neuron depolarization and an influx of calcium ions, eventually causing long-term synaptic depression mediated by endocannabinoids [31]. Figure 1 demonstrates the mechanism by which CBD affects psychotic disorders.

Cannabinoids have several effects on the central nervous system (CNS) which may contribute to their efficacy in the management of pain [32].

## 4. Cannabinoids and Analgesia

As for pain management and the effect of the endocannabinoid system on pain and proprioception, it is a matter of debate which formulation of cannabinoids should be used [11]. THC is considered inferior to synthetic cannabinoids in the management of pain, since it is non-selective and has a wider spectrum, activating both CB1 and CB2 receptors [11]. As mentioned above, cannabinoids have an analgesic effect due to their inhibition of nociceptive stimuli [11]. CB1 receptors are abundant in areas such as the spinal cord, thalamus, and dorsal root ganglia, which help relay nociceptive stimulation. Cannabinoids like THC can bind cannabinoid receptors. Cannabinoids have an analgesic effect due to their inhibition of nociceptive stimuli, impeding their activity and decreasing nociceptive information migration [11]. Recent studies reaffirm the efficacy of cannabinoids in managing pain and inflammation. A 2024 review discussed the dual anti-inflammatory and analgesic properties of THC and CBD. These effects are mediated through actions on peroxisome proliferator-activated receptors (PPARs) and transient receptor potential vanilloid 1 (TRPV1), alongside the inhibition of cyclooxygenase enzymes, which blocks inflammatory mediators at the site of injury [33]. Furthermore, Jylkkä et al. conducted a study on chronic pain patients which suggested that THC can enhance the analgesic effects of opioids, potentially reducing the required dose of opioids without compromising pain relief. This synergy supports the development of combination therapies for chronic pain management [34].

## 5. Forms of Cannabis and Administration Routes

In the emerging efforts to discuss the use of cannabis and cannabinoids for medicinal purposes, it is of paramount importance to mention the various forms in which cannabis may be administered, while discussing the efficacy, advantages and disadvantages of such forms and routes of administration. Various forms of cannabis include smoking, vaping, edibles, dabbing (the inhalation of concentrated vapors for a stronger effect), Butane hash oil, and topical ointments [35,36]. The most common method of consumption remains smoking, while other forms are rapidly rising in incidence.

Inhalation methods of consuming cannabis, including smoking, vaping, and dabbing, may have adverse respiratory side effects. Cannabis is considered a risk factor for respiratory disease and may cause a decline in pulmonary function test results when smoked for a long time, and particularly a reduction in the forced expiratory volume in 1 s (FEV1) [37]. Smoking cannabis is also considered a prominent risk factor for increasing the likelihood of bullous lung disease, spontaneous pneumothorax, and hypersensitivity pneumonitis [38].

Oral formulations seem to have milder and safer effects, since they avoid respiratory effects and a decline in pulmonary function, as well as being variable in efficacy due to differences in metabolism between different preparations, as some preparations do not even include THC [35,39]. However, due to the delayed effect of oral compared to smoked cannabis, there is a concerning risk of over-intoxication, which is generally avoided in smoking due to reaching a faster “high”. Other risks include psychosis, which appears to be more common in oral than inhaled cannabis, as well as child poisoning due to the accidental ingestion of oral preparations [40].

As for topical ointments, Xu et al. [41] conducted a randomized and placebo-controlled trial where 29 patients with chronic neuropathic pain were divided into two groups. One group was given a CBD-containing ointment. The other was administered a placebo. The results indicated a significant decrease in sharp and intense pain in the CBD group, as well as a decrease in unwanted or unpleasant sensations, such as cold or itchiness. Moreover, there were no adverse effects reported during this four-week trial, suggesting that CBD-containing ointments might be a promising choice for the management of chronic neuropathic pain. Additionally, topical CBD has been reported to cure some skin diseases, mostly due to its anti-inflammatory and immunosuppressive effects, which help reduce and manage symptoms of immune-mediated inflammatory skin conditions [42].

## 6. Adverse Effects and Safety Concerns

### 6.1. Overview

Medicinal cannabis is commonly used by patients to treat a variety of illnesses and symptoms, such as chronic non-cancer pain [43]. No systematic assessment of the safety of cannabis use for medical purposes has been conducted [44]. In this article we collect the adverse effects of cannabis, including both short- and long-term effects.

In many nations, cannabis stands out as it is the most commonly used illegal substance, because there is conflicting information regarding many of the impacts of cannabis use. We have condensed the probable adverse health and psychological outcomes of both short-term symptoms, including memory loss, coordination issues, and impaired decision-making, and long-term symptoms such as cannabis dependency, cognitive decline, and chronic bronchitis [43,45]. It has been shown that both short-term and long-term cannabis usage can have negative effects on memory, motor coordination, paranoia, psychosis, and cognitive function and increase the risk of anxiety and depression [44]. The incidence of adverse events of cannabis use is correlated with gender and past cannabis use. In one study, 1273 adverse events were reported, with females who had never used cannabis before and those who had stopped using it both having higher odds of experiencing adverse events [46].

### 6.2. Acute Intoxication

A study by Hyman et al. [47] investigated the stress-related factors of cannabis use; the study concluded that the administration of THC in healthy individuals produced psychotic symptoms, altered perception, increased anxiety, and cognitive deficits. In terms of acute toxicity, there is no proof that consuming cannabis can result in overdose or fatal circumstances [48].

Acute intoxication can cause euphoria, perception alterations such as time and spatial distortion, the intensification of ordinary sensory experiences, and motor impairment [49]. The amount and length of exposure determine the intoxication of cannabis, while the absorption is determined by the concentration and route of delivery. Cannabis consumption by the airway is the most common consumption method, including smoking and vaporization [50]. Plasma concentrations of THC and CBD are detected within seconds of inhalation, peaking 3 to 10 min later [51]. THC has a strong lipophilicity, which leads to serum concentrations peaking within 15 to 30 min and lasting up to 4 h [24]. The individual host’s metabolism, puff duration and numbers, inhalation volume, the size of particle, and inhalation device can all affect the peak plasma concentration and the time it takes to reach the peak [51,52]. While the benefit of ingested cannabis is that the number of harmful chemicals produced during ingestion is lower compared to smoking [51,53], oral use has a delayed onset of action in comparison to inhalation; peak concentrations of THC from oral consumption typically occur within 1 to 2 h and last up to 8 h. The duration of its psychoactive effects is protracted, with a 3 to 30 h delayed return to baseline and a 12 h mean duration [54,55]. Oromucosal delivery is an additional oral administration technique that is applied therapeutically. Higher plasma concentrations are produced by faster absorption through the buccal mucosa as opposed to the ingestible delivery route [56]. The sublingual administration of medication avoids the first-pass hepatic metabolism [53]. As of right now, no fatalities from acute or direct poisoning have been documented. On the other hand, inadvertent ingestion by children has often resulted in comas [44].

There is limited certainty in the evidence indicating that adverse effects are common in patients using medical cannabis or cannabinoids for chronic pain. However, it appears that severe adverse events are infrequent among patients. These adverse events may lead to a discontinuation of usage, cognitive adverse events, accidents and injuries, and dependency and withdrawal symptoms [46].

### 6.3. Acute and Chronic Effects

Regarding the effects of cannabis on children and adolescents, research indicates that the developing teenage brain seems to be more susceptible to the long-term effects of marijuana usage [57]. Long-term cannabis use, particularly when it begins in adolescence, may be linked to memory loss and other cognitive impairments, including a drop in IQ test results [57]. Additionally, adolescent marijuana usage appears to be linked to a higher prevalence of schizophrenia/psychosis, especially in those with a genetic predisposition [58].

Several acute and chronic health problems associated with cannabis use have been identified, including central nervous system effects. Patients should be aware that the initial response to cannabis use varies based on factors like genetic predisposition and the amount of THC present. Euphoria and relaxation are typical immediate psychological responses to cannabis, which is why studies indicate that relaxation and stress management are primary reasons for its medical use [47,59]. However, anxiety, dysphoria, and psychosis or hallucinations may occur as immediate effects [60]. Additional possible acute effects could cause alterations in the way that colors, noises, and time are perceived, as well as cognitive abnormalities like poor judgment, poor memory, inattentiveness, and impaired coordination [61]. Other acute effects include acute physiological effects, most notably tachycardia, conjunctival redness, increased appetite, and dry mouth. The diagnostic criteria for acute cannabis intoxication include the presence of two or more of these physical findings [62].

Additionally, acute cardiovascular adverse effects that should be considered include tachycardia probably via direct agonism of the CB1 receptors in cardiac tissue [63]; an increase in cardiac output, an elevation of blood pressure, postural hypotension, vasodilation (flushing, red eyes), and cardiac toxicity may occur via additive hypertension and tachycardia with amphetamines, cocaine, atropine or other sympathomimetic agents [64]. These symptoms are often observed within one hour of inhaling THC [63]. These effects could lead to an elevation in myocardial oxygen demand. Studies have indicated a potential correlation between this effect and the precipitation of angina and myocardial infarction within one hour of use [64,65]. Consequently, marijuana may thereby increase the risk of cardiovascular disease, particularly in those with a history of cardiovascular illness.

Regarding chronic effects, central nervous system impairment is notable. The brain is most affected by long-term cannabis use, particularly when usage starts in adolescence, because adolescent cannabis usage, when compared to starting use as an adult, is associated with an increased risk of developing psychosis, leading to long-term negative effects such as cannabis use disorder, memory loss, and other cognitive impairments [57,66,67]. According to a recent research, adults who use cannabis regularly have a higher risk of developing psychosis, and the risk rises with increased cannabis consumption [68].

Furthermore, chronic pulmonary effects, especially if cannabis is smoked, are also noteworthy. Cannabis smoking was found to have a higher risk of respiratory immunological impairment as well as a higher risk of respiratory infections, bronchitis, and chronic obstructive pulmonary disease [38]. Long-term marijuana usage is linked to airway inflammation and chronic bronchitis, much like long-term tobacco smoking [45,69]. However, low-level marijuana use over a long period of time did not result in a decrease in pulmonary function testing, in contrast to that seen with tobacco smoking [70]. While carcinogenic substances are present in both cigarette and cannabis smoke, a recent international study found that there is no significant increase in the risk of lung cancer among chronic marijuana users [71].

### 6.4. Cannabis and Addiction

Addiction Risk and Dependency Concerns: While cannabis, particularly THC, shows promise as an alternative to opioids in managing chronic pain, there is an important consideration regarding the risk of dependency. Chronic use, especially of THC, can lead to cannabis use disorder (CUD), marked by tolerance, compulsive use, and withdrawal symptoms. Although the dependency potential of cannabis is generally considered lower than that of opioids, prolonged use and high doses can increase the likelihood of misuse. Patients with a history of substance abuse or those using cannabis for extended periods may be at higher risk. Healthcare providers should monitor for signs of misuse and educate patients about the potential risks of dependency. Additionally, more research is needed to fully understand the long-term effects of cannabis use on dependency and to create evidence-based guidelines that balance the benefits of pain relief with the risks of addiction [72].

### 6.5. Contraindications

There are several recommended contraindications to, and cautions about, the use of medical cannabis. Contraindications to cannabinoid therapies include significant psychiatric, respiratory, cardiovascular, renal, or hepatic illness [73]. Smoked cannabis is contraindicated in some cases such as in patients who are 25 years old or younger; people who have a current, past, or strong family history of psychosis and schizophrenia; people who have a current or history of cannabis use disorder; people who have a current substance use disorder; people who have cardiovascular or respiratory disease; or people who are pregnant or planning to become pregnant or breast-feeding [74].

It should be used with caution in patients who are tobacco-smoking or alcohol users, who are at an increased risk of cardiovascular disease, who have anxiety or mood disorders, or who are taking higher doses of opioids or benzodiazepines [74].

## 7. Medical Effectiveness of Cannabinoids in the Treatment of Chronic Non-Cancer Pain

### 7.1. Overview

Chronic pain is any painful condition that lasts over 3 months and is associated with a worse quality of life compared to other chronic heart, lung, and kidney diseases [75]. This includes numbing, neuropathic, and centralized pain [76,77]. Numerous scales are used in the assessment of chronic pain outcomes, including the visual analog scale, the numerical rating scale, the Profile of Mood States (POMS) Questionnaire, the 36-item short-form survey (FS36), the Neuropathic Pain Scale, and the McGill Pain Questionnaire [78]. Notably, some of these evaluation instruments emphasize functionality, quality of life, and patient-reported outcomes.

Taking care of chronic pain and its co-occurring morbidities like sleep disorders, anxiety, depression, post-traumatic stress disorder, and substance use disorders like opioids and alcohol use disorders is a medical challenge [79,80,81,82,83,84,85]. Research shows that 19–29% of Canadians who are 18 years or older have suffered from chronic pain for more than 10 years. Chronic pain is typically related to arthritis and lower back pain [86]. Over 4.2 million Canadians suffer from chronic pain and this number is predicted to increase by 2031 [87]. These data are similar in several other countries.

Notably, chronic pain and its concurring conditions rank among the most common conditions for which cannabinoid-based products are utilized for therapeutic purposes [76,88,89,90]. Evidence of moderate quality suggests that nabilone, THC/CBD pills (not FDA-authorized), nabiximols (not approved in the US), and dronabinol (not approved) may be beneficial for treating spasticity in multiple sclerosis. Additionally, nabilone, nabiximols, and smoked THC (not allowed by the FDA) may be beneficial for treating neuropathic pain or chronic cancer pain.

There is no advantage to using nabiximols to treat depression, according to extremely low-quality research [91]. There are not enough data to draw any firm conclusions about glaucoma treatment, as only one study with six participants was evaluated [91].

### 7.2. Cannabis and Neurological Conditions

The 2013–2014 Cochrane Reviews concluded that there is not enough evidence to support the use of cannabinoids to treat schizophrenia [92], seizures/epilepsy [93], HIV/AIDS [94], or cannabis use disorder [95]. For the treatment of diabetes and metabolic syndrome, a plant extract combination including the cannabinoids CBD and tetrahydrocannabivarin is being investigated [96]. Tetrahydrocannabivarin may provide potential benefits for the management of type 2 diabetes mellitus, according to a recent systematic review [97]. An epidemiologic study that found a correlation between marijuana usage and a smaller waist circumference, lower fasting insulin levels, and decreased insulin resistance [98], which supports the use of marijuana in the treatment of diabetes and metabolic syndrome.

Between 2003 and 2010, a review of 18 trials involving 766 individuals examined the effectiveness of cannabis medications in treating pain, particularly neuropathic pain. The trials were of high quality, with 15 trials showing substantial analgesic effects and improvements in sleep quality [99,100,101,102,103]. The treatments were compared with placebo and smoked cannabis, with no significant side effects observed. The mean treatment length was 2.8 weeks, with mild effects and minimal adverse effects [104].

This comprehensive analysis of eighteen recent high-quality randomized studies shows that, when it comes to treating chronic non-cancer pain (often neuropathic pain), cannabinoids are a safe and somewhat effective treatment option. There is an urgent need for more treatment alternatives due to the prevalence of chronic pain, its impact on function, and the lack of effective therapeutic approaches. More extensive studies with longer durations that report on pain and function level are needed [104].

However, there are other studies, like one 4-year prospective cohort study, that aim to investigate the clinical effectiveness of cannabis in managing chronic non-cancer pain and its potential role in reducing opioid dose requirements [105]. Participants in this study had chronic non-cancer pain and were receiving opioid treatment. The study, involving 1514 participants, found that cannabis use was common for pain relief, with 24% using it for pain. Interest in cannabis usage increased from 33% to 60% within the four years. Participants who used cannabis had higher pain severity and interference, and lower pain self-efficacy and generalized anxiety disorder severity scores. There was no evidence of a temporal relationship between cannabis use and pain severity and no evidence of reduced opioid use or opioid discontinuation. Large clinical trials are needed to determine the efficacy of cannabis for chronic non-cancer pain.

In randomized clinical trials comparing medical cannabis to any non-cannabis control for chronic pain at more than one month of follow-up, results showed moderate- to high-certainty evidence that non-inhaled medical cannabis or cannabinoids result in a small-to-very-small improvement in pain relief, physical functioning, and sleep quality among patients with chronic pain [106].

### 7.3. Patient Perspectives and Quality of Life

The medical community’s approach to medicinal cannabis remains cautious. According to a survey of Australian GPs, many feel inadequately informed about access pathways, available products, and the evidence supporting medicinal cannabis [106]. While patient inquiries are common, only a small percentage of doctors feel comfortable discussing cannabis with them. However, overall, GPs show positivity towards prescribing medicinal cannabis, particularly for serious conditions like cancer pain, chemotherapy-induced nausea and vomiting, epilepsy, and difficult-to-treat neurological conditions, provided they receive sufficient education [107]. Specialist colleges and the Australian Medical Association maintain conservative stances in the medicinal cannabis debate due to concerns about limited evidence from clinical trials and potential adverse effects [107].

Recent studies have shown the efficacy of medicinal cannabis in chronic pain conditions such as osteoarthritis, neuropathic pain, and other non-cancer chronic pain. However, there are limitations regarding dosage, treatment duration, adverse effects, long-term follow-up, and dependence that require further investigation, especially concerning fibromyalgia pain [108].

Despite these limitations, there is hope that cannabis and cannabinoids could provide relief for those suffering from chronic pain associated with fibromyalgia, but the potential benefits must be weighed against possible harmful effects [108].

The findings on the efficacy of cannabis for chronic non-cancer pain (CNCP) are inconclusive. While some studies suggest that inhaled cannabis can improve symptoms associated with CNCP, its effectiveness may vary depending on factors like dosage and the route of administration. Moreover, the lack of appropriate placebo controls in some trials may lead to an overestimation of the benefits [109].

Patients using medicinal cannabis for chronic non-cancer pain perceive more benefits than harms, as indicated by studies. Additionally, research shows promising results regarding the use of nabiximols in managing advanced pain and reducing opioid use over time [110].

However, patients using THC-containing products should be cautious about driving or operating heavy machinery, even after waiting at least six hours post-consumption, as they remain vulnerable to legal prosecution. Sudden withdrawal from cannabis can also cause mild sleep and appetite disturbances, cravings, and emotional changes, which should be considered [107].

### 7.4. Comparative Analysis with Other Pain Management Options

Recent evidence indicates that approximately 16% of CNCP patients use both cannabis and prescription opioids. Furthermore, population health research has identified a correlation between the legalization of medical cannabis and reductions in opioid-related deaths, hospitalizations, and overdoses, along with decreased expenditures associated with prescription opioid overdose and misuse [109].

Patients have expressed interest in medical cannabis as a possible adjunct to or substitute for opioid pain medication [109]. Moreover, a significant observational study involving cancer patients who used cannabis over a span of six months revealed a decrease in the number of patients experiencing severe pain, as well as a reduction in opioid usage [110].

## 8. Future Directions in Research and Clinical Practice

At the same time, research indicates that the prevalence of cannabis use has increased in recent years, with cannabis being the most commonly used illicit drug in the United States [109].

For Canadians, the role of cannabis-based products (CBPs) derived from the cannabis plant in managing chronic pain and its associated co-occurring conditions is expected to expand. However, integrating CBPs into the therapeutic arsenal poses a complex challenge for public health and medical practitioners [111].

However, more research is needed on how the cannabinoid system interacts with the pain pathway [108]. Further investigation is necessary to understand the interactions between the cannabinoid system and the pain pathway. Cannabinoid receptors, namely CB1 and CB2, play a key role in mediating the analgesic effects of cannabinoids, primarily through the inhibition of pre-synaptic gamma-aminobutyric acid (GABA) and glutamatergic transmission. CB1 receptors are primarily located in the central nervous system (CNS), while CB2 receptors are mostly found outside the CNS. The activation of these receptors is believed to have both anti-nociceptive and anti-inflammatory effects on chronic pain conditions [108].

Although many cannabinoids have been identified, tetrahydrocannabinol (THC) and cannabidiol (CBD) are the most clinically relevant components. THC primarily influences pain perception, appetite, orientation, and mood, while CBD exhibits anti-inflammatory, anti-anxiety, and analgesic properties. Notably, THC acts as a partial receptor agonist, whereas CBD functions as a negative allosteric modulator of the CB1 receptor [108].

Further research, including extended studies, is warranted to evaluate the long-term efficacy, adverse effects, and potential for dependence of cannabinoids. Conducting more clinical trials with extended follow-up periods and investigating cannabinoids’ dose–response relationship are crucial in advancing our understanding of this therapeutic modality [108].

## 9. Conclusions

This review presents a comprehensive synthesis of the current evidence on the use of cannabis in managing chronic non-cancer pain, a condition that significantly impacts quality of life and often coexists with psychological disorders. Unlike many previous reviews that concentrate on either individual cannabinoids or specific consumption methods, this manuscript provides a comprehensive overview of both THC and CBD, examining their combined effects and the impact of different administration routes on pain management and quality of life. By considering patient perspectives, regulatory frameworks, and the potential adverse effects of cannabis, this review offers a nuanced approach to chronic pain management that is grounded in both clinical practice and evolving regulatory landscapes. Furthermore, it highlights the gaps in long-term safety and efficacy data, reinforcing the need for future studies to establish comprehensive guidelines for cannabis use in pain management. This distinction helps to shed light on an emerging area of research, offering a broader and more integrative view compared to earlier works. Despite the comprehensive nature of this review, there are several limitations that should be considered. First, the majority of the studies discussed are observational or had short-term follow-ups, which limits our ability to draw definitive conclusions about the long-term safety and efficacy of cannabis for chronic non-cancer pain. Additionally, the heterogeneity in the study designs, including variations in dosage, formulation, and patient populations, introduces challenges in comparing results across studies. This review also reflects the current state of the evidence, which is still evolving, and thus some recent studies or emerging data may not be included. Lastly, the regulatory and legal landscape surrounding cannabis use varies greatly across different regions, and this review may not fully account for all regional differences in prescription practices or patient access, which can influence outcomes. These limitations highlight the need for further research to address gaps in our knowledge and clarify the role of cannabis in chronic pain management.
